# Fatal neurotoxic envenomation following the bite of a greater black krait (*Bungarus niger*) in Nepal: a case report

**DOI:** 10.1186/s40409-016-0073-8

**Published:** 2016-06-03

**Authors:** Deb Prasad Pandey, Sanjib Kumar Sharma, Emilie Alirol, François Chappuis, Ulrich Kuch

**Affiliations:** Kaligandaki Health Foundation, Kawasoti, Nawalparasi Nepal; Department of Internal Medicine, B.P. Koirala Institute of Health Sciences, Dharan, Nepal; Clinical Trial Unit, Clinical Research Centre and Division of Tropical and Humanitarian Medicine, University Hospitals of Geneva, Geneva, Switzerland; Médecins Sans Frontières UK, London, UK; Institute of Occupational Medicine, Social Medicine and Environmental Medicine, Goethe University, Frankfurt am Main, Germany

**Keywords:** *Bungarus niger*, Neurotoxicity, Krait, Envenomation, Antivenom, Snakebite

## Abstract

**Background:**

Neurotoxic envenomation following bites by kraits (*Bungarus* species) is a leading cause of snakebite mortality in South Asia. Over a long time, this had been attributed only to one species, the common krait (*Bungarus caeruleus*). However, recent research has provided increasing evidence of the involvement of several krait species. Here, we report a fatal case of neurotoxic envenomation following the bite of a greater black krait (*Bungarus niger*) in Nepal.

**Case presentation:**

A 33-year-old man was bitten in the outdoor corridor of his home in the eastern hills of Ilam district while handling a snake he thought to be non-venomous. He subsequently developed severe abdominal pain, frequent vomiting, and signs of neurotoxic envenomation leading to respiratory paralysis. The patient did not respond to Indian polyvalent antivenom given 4 h after the bite and died under treatment 8 h after the bite. This is the second time that a *B. niger* was observed in Nepal, the first documented case of envenomation by this species in the country and the sixth reported case worldwide.

**Conclusions:**

Previous distribution records – from eastern India and western Nepal, from western hills in Nepal, and from lowland localities in India and Bangladesh – indicate risk of envenomation by *B. niger* throughout the low and intermediate elevations of Nepal up to at least 1,500 m above sea level. As very few people in Nepal bring killed snakes to healthcare centers and because there is a general belief among local people that there are no kraits in the hills, bites by *B. niger* are likely to be misdiagnosed and underreported.

## Background

For a very long time, krait bite envenomation in South Asia was thought to be due to a single species, the widely distributed common krait (*Bungarus caeruleus*), whose venom has consequently been used in the production of polyvalent antivenoms in India [1]. However, several other species of krait occur in South Asia, and some of these were recently shown to contribute to regional snakebite morbidity and mortality [2–7].

**Fig. 1:**
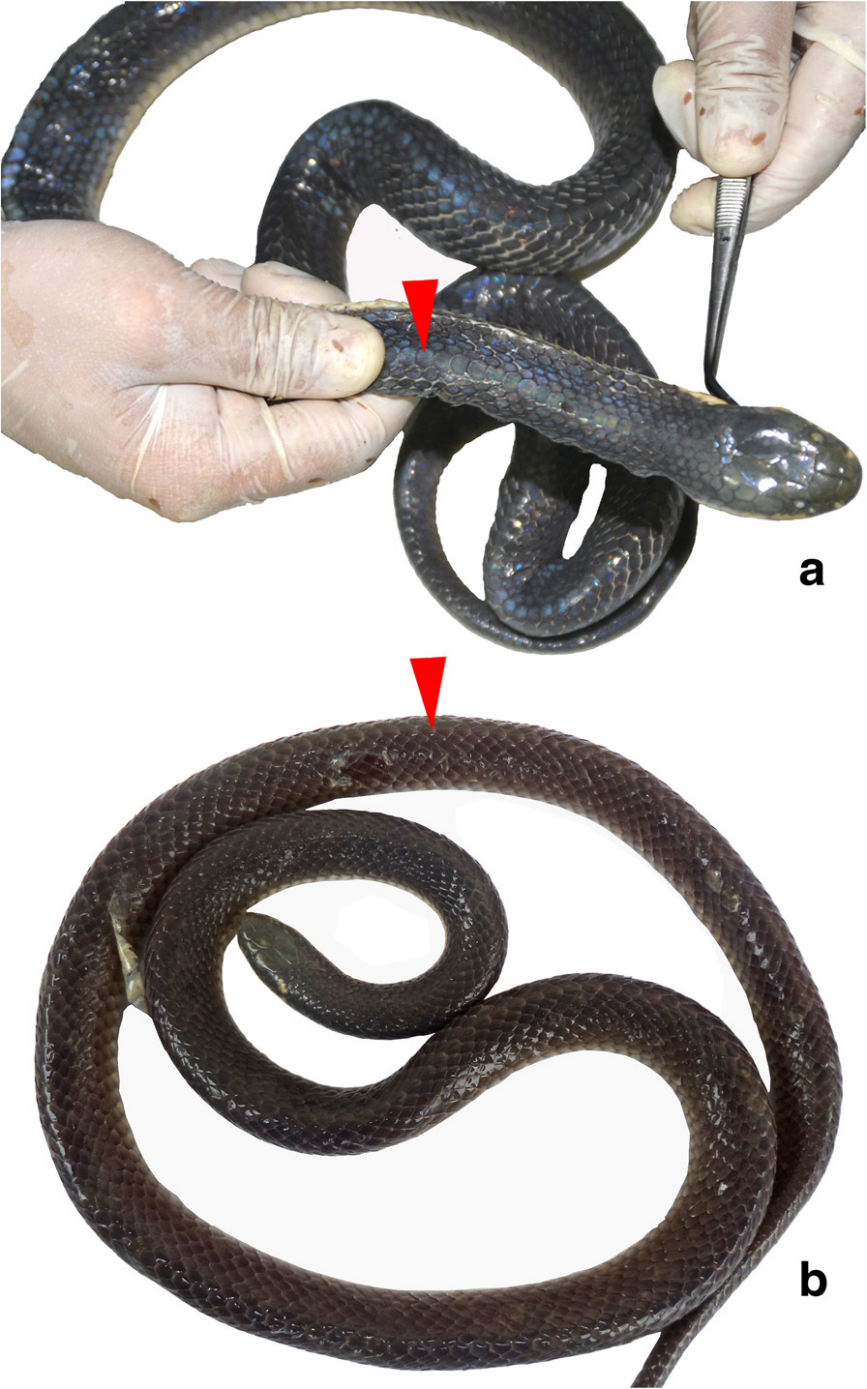
Dorsal view of **a** *Bungarus niger* and **b** *Bungarus lividus*. The entirely black dorsum of both species distinguishes them from other South Asian kraits. In (**a**) *B. niger* the middorsal scales along the vertebral column (red arrows) are greatly enlarged and hexagonal in shape, whereas in (**b**) *B. lividus* the middorsal scales are similar in size and shape to those of adjacent scale rows (photo by Deb Prasad Pandey)

The greater black krait (*Bungarus niger* Wall, 1908) is widely distributed in South and Southeast Asia where it has been recorded in India, Bangladesh, Bhutan, Myanmar, and Nepal [2, 4, 8–10]. *Bungarus niger* is superficially similar in appearance to the lesser black krait (*Bungarus lividus*) (Fig. 1) which has caused fatalities in India and Nepal [5]. However, *B. niger* is distinguishable from *B. lividus* based on the size and shape of the middorsal row of scales (Fig. 1). Although *B. niger* has been recognized as a distinct species for more than a century, only a single series of five confirmed cases of envenomation by this species in Bangladesh has been published [4, 11]. Strikingly, *B. niger* was found to cause both neurotoxic and myotoxic envenomation in victims as well as in animal models, a new syndrome of snakebite envenomation in South Asia that entails additional clinical challenges [5]. Available antivenoms in this region, produced by various Indian manufacturers, are raised against only four species of venomous snakes, namely, common krait (*B. caeruleus*), spectacled cobra (*Naja naja*), Russell’s viper (*Daboia russelii*), and saw-scaled viper (*Echis carinatus*) [1]. Whether or not these or other antivenoms can neutralize the lethal effects of *B. niger* venom is still unknown. In the present study, we present the first case of *B. niger* envenomation in Nepal.

## Case presentation

A 33-year-old male laborer found a snake on the outdoor corridor of his house at Golbasti, Ilam municipality, Ilam district, in the eastern hills of Nepal (N 26.89874°, E 87.91900°, elevation 1515 m a.s.l.) at 6:30 p.m. on 4 August 2012. Believing that it was a non-venomous snake, he picked it up and carelessly handled the snake. The victim, who had been under the influence of locally produced alcoholic drink (“*Jaand*”), hit the back of the snake once with a wooden plank and then picked the snake up again and played with it joining its head with the tail. When his wife urged him not to handle the snake, he replied that the snakes in the hills of Nepal were non-venomous. Unresponsive to advice, the man touched and pressed the injured back of the snake. In this moment, at 7:00 p.m., the snake bit the index finger of his right hand. The man then picked the snake up and put it in a bucket.

After the bite, the patient’s brother applied a tourniquet to his forearm. The patient then walked for about 10 min to his neighbor’s house. The neighbor immediately carried him on a motorcycle to the hospital in Ilam Bazaar (Ilam district, Nepal, distance 2.2 km) where he arrived at 7:30 p.m. On admission, there were fang marks indicating multiple bites with slight bleeding from the bite sites. Healthcare staff washed the finger, applied a crepe bandage, and infused saline over 30 min. At 8:00 p.m., the patient was suffering from burning sensation at the bite site and appeared significantly ill, he had developed bilateral ptosis, difficulty in speaking, slurred speech, and had pain in the chest and abdomen. Moreover, he vomited for the first time. As antivenom was not available in this hospital, arrangements were made to transfer the patient to the snakebite treatment center of Charali in Jhapa district. At 8:30 p.m., the ambulance went from Ilam to Charali with the patient. At 9:00 p.m., difficulty in speaking increased so the patient started to communicate by nodding his head, shaking his hands and writing something with his index finger which his illiterate wife could not understand. He also vomited for a second time and, at 9:15 p.m. and 10:30 p.m., a third and fourth time. At 10:50 p.m., when the ambulance reached Charali, the patient was semi-conscious.

On admission to the Charali Snakebite Treatment Center (about 4 h after the bite), the attending healthcare staff were not able to find any fang marks, presumably as an effect of the bite site having been washed at Ilam Hospital. There was no detectable swelling or inflammation. However, bilateral ptosis, difficulty in swallowing, slurred speech, and difficulty in speaking were recorded at 11:00 p.m. Antivenom was administered at 11:15 p.m. in an initial bolus dose of 100 mL of polyvalent anti-snake venom serum (produced by VINS Bioproducts Ltd., targeted against *B. caeruleus*, *N. naja*, *E. carinatus,* and *D. russelii* snake venoms) preceded by a subcutaneous injection of adrenaline (0.25 mL of 1:1000 solution) [12]. Atropine (0.6 mg) and neostigmine (0.5 mg) were given with the same infusion as an attempt of anticholinesterase treatment. At 11:20 p.m., the patient vomited for the fifth time and received antiemetic treatment in the form of an intravenous injection of metoclopramide (10 mg). At 11:35 p.m., the patient showed rashes near the infusion cannula at the left hand and was given an intravenous injection of pheniramine maleate (10 mg). The same dosage of atropine and neostigmine treatment was repeated at 11:45 p.m., although the first injections had not resulted in any improvement.

At 00:05 a.m. on 5 August 2012, the following symptoms were noted: the patient was unable to speak, could not protrude the tongue, swallow, or frown. A second dose of 100 mL of antivenom was injected and his airways cleared using a suction pump. At 00:15 a.m. he was again given atropine and neostigmine (0.6 and 0.5 mg, respectively). At 00:25 a.m., the patient was re-evaluated and found to show no improvement. At 00:30 a.m., he received a third dose of 100 mL of antivenom by slow intravenous bolus injection. By this time, the patient’s respiratory rate was altered, his blood pressure lowered, and blood oxygen level decreased. Attempts were made to intubate the patient but failed three times. Then, attempts were made to manually ventilate him with a mask and Ambu bag. When his pulse rate decreased to 55, he was given atropine (0.6 mg, subcutaneously) which increased the rate. At 00:45 a.m., endotracheal intubation was successful, but the patient’s blood oxygen level was down to 40 % and his blood pressure dropped, immediately followed by cardiac arrest. Cardiopulmonary resuscitation was attempted until he was declared dead at 01:00 a.m. (8 h after the bite). Because of the absence of laboratory facilities in the Charali Snakebite Treatment Center, no information on the patient’s ECG, potassium and sodium levels, total leucocyte count, blood urea, creatine kinase, albumin, myoglobin or blood cells in urine was available.

The snake specimen brought to the Charali Snakebite Treatment Center by the snakebite victim was a male *B. niger* measuring 1,357 mm in total length. The snake was labelled and preserved in 70 % ethanol. After morphological examination and taxonomic identification by one of the authors (DPP), it was permanently deposited in the snake collection of the B. P. Koirala Institute of Health Sciences, Dharan, Nepal (voucher number 02-023).

## Discussion

This is the first report of *B. niger* bite envenomation in Nepal, and only the second record of this species from this country. This case report highlights the diversity of snake species causing fatalities in Nepal, and emphasizes the need of preserving snakes brought by snakebite victims to better understand snakebite epidemiology. In addition, it corroborates recent publications describing previously overlooked snake species that contribute to snakebite mortality in South Asia [1, 4, 13].

Including the present one, six proven cases of *B. niger* envenomation with three fatalities have been reported since these snakes had first been recognized as a distinct species, in the year 1908 [11]. Five of the bites occurred in Bangladesh [4]. The known altitudinal distribution of this species ranges from near sea level in Bangladesh up to at least 1,500 m in Nepal [4, 8, 14]. As there are records of this species from India, western and eastern Nepal, and two records in the mid-western and eastern hills of Nepal (the present one), *B. niger* may be expected to occur throughout the latter country [8, 10, 15, 16]. Moreover, it may be potentially found at lower and higher altitudes than those of the two presently known collecting sites (1,450 and 1,515 m). In Bangladesh, *B. niger* inflicted bites (*n* = 3) while being handled (like the present case), on sleeping people (*n* = 2), and on a person who was cutting wood [4]. Similar to *B. caeruleus* bites*, B. niger* bites occurred in and around houses in the evening (present case), at night (*n* = 1, 2:00 a.m.), and in the morning (4:00 to 9:00 a.m.) [4, 17–19]. Therefore, people should be careful while performing household and field activities especially at twilight and night during the rainy season (July-August) [10].

Most of the community people we have talked to in the highlands of Nepal believe that the deadly venomous nocturnal kraits are absent in the hills. The victim, his family members, and other locals did not know that this snake was venomous. Tillack and Grossmann [10] reported a similar ignorance of this species among inhabitants of the western hills of Nepal when they first recorded *B. niger* there. In the present case, the snake apparently was quite docile initially and did not attempt to bite until it was seriously injured. This kind of inoffensive behavior, typical of most kraits at least during daytime, may be misleading villagers who think that these snakes are not dangerous.

Although the *B. niger* bite occurred in a place connected by a gravel (0.5 km) and paved road (74 km) with the nearest Snakebite Treatment Center in Charali, Jhapa district, accessible by a four-wheel vehicle within 10 to 30 min, the patient lost precious time (about 1 h) getting to the local health facility where no antivenom was available. This highlights a need for informing people about the nearest healthcare facility that delivers adequate treatment for snakebite envenomation and has antivenom (a WHO recognized ‘essential drug’) on stock.

In contrast to the *B. niger* bite cases in Bangladesh, we could not evaluate the possibility of myotoxic envenomation and ensuing complications in this patient (i.e., rhabdomyolysis, myoglobinuria, hyperkalemia, acute renal failure, etc.) because of the absence of laboratory facilities [4]. We noted symptoms of burning sensation at the bite site, bilateral ptosis, abdominal pain, and frequent vomiting similar to *B. caeruleus* and *B. lividus* envenomation [5, 17, 18].

In the Bangladesh series of *B. niger* envenomation, three of the five patients recovered completely after about 3 to 10 days (two of these requiring mechanical ventilation and treatment with antivenom), and two died within 47 h after the bite due to respiratory paralysis and acute renal failure [4]. Collectively, these observations indicate delayed or no recovery after envenomation, despite the administration of Indian polyvalent antivenoms. The efficacy of the currently available antivenoms in the treatment of *B. niger* envenomation is therefore doubtful.

## Conclusions

Although only six proven cases of envenomation by *B. niger* have been published from Bangladesh and Nepal, more fatalities due to the bite of this species can be expected to occur in a large region of South Asia. Epidemiological studies aimed at identifying the snake species responsible for bites should therefore be conducted. Community-based health education, highlighting simple messages on snakebite prevention and rapid transport of victims to appropriate medical care, should be reinforced. Health care workers in snakebite treatment centers and ambulances in South Asia and beyond should be trained to perform intubation and assisted ventilation as a priority, as currently available antivenoms are unlikely to be effective against envenomation by *B. niger* (and probably other *Bungarus* species). Furthermore, methods of preventing and treating acute renal failure –as an additional possible clinical challenge after bites by this and other snake species– should be included in training programs [20].
